# The Ecology of Exercise: Mechanisms Underlying Individual Variation in Behavior, Activity, and Performance: An Introduction to Symposium

**DOI:** 10.1093/icb/icx083

**Published:** 2017-07-21

**Authors:** Shaun S. Killen, Ryan Calsbeek, Tony D. Williams

**Affiliations:** * Institute of Biodiversity, Animal Health and Comparative Medicine, University of Glasgow, Graham Kerr Building, Glasgow, G12 8QQ, UK; † Department of Biological Sciences, Dartmouth College, Hanover, New Hampshire 03755, USA; ‡ Department of Biological Sciences, Simon Fraser University, Burnaby, British Columbia V5A 1S6, Canada

## Abstract

Wild animals often engage in intense physical activity while performing tasks vital for their survival and reproduction associated with foraging, avoiding predators, fighting, providing parental care, and migrating. In this theme issue we consider how viewing these tasks as “exercise”—analogous to that performed by human athletes—may help provide insight into the mechanisms underlying individual variation in these types of behaviors and the importance of physical activity in an ecological context. In this article and throughout this issue, we focus on four key questions relevant to the study of behavioral ecology that may be addressed by studying wild animal behavior from the perspective of exercise physiology: (1) How hard do individual animals work in response to ecological (or evolutionary) demands?; (2) Do lab-based studies of activity provide good models for understanding activity in free-living animals and individual variation in traits?; (3) Can animals work too hard during “routine” activities?; and (4) Can paradigms of “exercise” and “training” be applied to free-living animals? Attempts to address these issues are currently being facilitated by rapid technological developments associated with physiological measurements and the remote tracking of wild animals, to provide mechanistic insights into the behavior of free-ranging animals at spatial and temporal scales that were previously impossible. We further suggest that viewing the behaviors of non-human animals in terms of the physical exercise performed will allow us to fully take advantage of these technological advances, draw from knowledge and conceptual frameworks already in use by human exercise physiologists, and identify key traits that constrain performance and generate variation in performance among individuals. It is our hope that, by highlighting mechanisms of behavior and performance, the articles in this issue will spur on further synergies between physiologists and ecologists, to take advantage of emerging cross-disciplinary perspectives and technologies.

## Introduction

The past decade has seen a steep rise in research focussing on individual trait variability within animal species (Williams 2008; Biro and Stamps 2010; Sih et al. 2015). Although among-individual variation has been long-recognized as the raw material on which natural selection operates to shape evolutionary trajectories (Darwin 1859; Huntingford 1976), this surge in interest has examined the role of specific traits in evolutionary processes (Dingemanse and Réale 2005; van Oers et al. 2005; Wolf and Weissing 2012), trait covariation (Biro and Stamps 2008; Careau et al. 2008), and the mechanisms that allow trait variation to persist in wild populations (Wolf et al. 2007; Dingemanse and Wolf 2010; Stamps and Groothuis 2010). The majority of this work has focused on individual variation in behaviors, such as the tendency to take risks while foraging or measures of spontaneous activity, exploratory behavior, or sociability (Sih et al. 2004; Réale et al. 2007). There have also been important advances in the quantitative analysis of trait variation and the degree to which individuals show behavioral plasticity in response to varying environmental conditions (Dingemanse et al. 2009; Dingemanse and Dochtermann 2013). At the same time, albeit at a slower rate, a body of research has accumulated on individual variation in physiological traits, particularly aspects of endocrine signaling and energetics (Williams 2008; Koolhaas et al. 2010; Burton et al. 2011; Norin and Malte 2011, 2012). Until very recently, these two domains of work examining variation in behavior and physiology have remained largely separate and so knowledge of the mechanistic basis of behavioral variation has been elusive (Killen et al. 2013).

Over time, however, research has shifted toward being more holistic with proposed links between animal personality and metabolic demand, and an accompanying focus on quantifying individual variation in physiological traits (Careau et al. 2008; Careau and Garland 2012; Killen et al. 2013; Mathot et al. 2015). It is becoming more appreciated, for instance, that individual animals within species vary not only in the amount of activity they display (with associated energetic costs (Montiglio et al. 2010; Murchie et al. 2011)) but also in their physiological capacity for maximum rates of activity and high-intensity exercise (Chappell et al. 1999; Norin and Malte 2011; Killen et al. 2012, 2014; Kasumovic and Seebacher 2013; Auer et al. 2015; Metcalfe et al. 2016). An outstanding question is to what extent variation in the maximum capacity for physical activity, often a target of lab-based studies, is ecologically relevant and affects individual fitness (Metcalfe et al. 2016). It is also possible that individuals that are able to increase their performance capacity via training effects (physiological plasticity) or quickly recover from intense exercise may gain additional fitness benefits, but these possibilities have been largely unexplored, especially in free-living animals (Halsey 2016; Bidder et al. 2017). It is also notable that even where studies of behavioral variation have examined mechanisms (Biro and Stamps 2010; Killen et al. 2013), the focus has mainly been on energetics while the role of other physiological systems has been mostly overlooked. For example, the integrated physiological mechanisms that underlie variation in foraging behavior remain almost completely unknown (Maurer 1996; Williams 2012). This is surprising given the central role of the endocrine system in modulating costs associated with reproductive investment which typically involves increases in activity.

As is demonstrated repeatedly throughout this issue, the current integration of physiological and behavioral research is being facilitated by technological advances in bio-logging, telemetry, and the tracking of animal movements in natural environments (Gill et al. 2005; Egevang et al. 2010; Hawkes et al. 2011; Klaassen et al. 2011; Brown et al. 2013; Cooke et al. 2013b). These technologies are providing novel perspectives and data that allow us to examine the behavior and physiology of individual animals in a new light. The emergence and integration of technologies for collecting data on animal movements, physiological parameters, and environmental variables, often developed by researchers working in traditionally disparate fields, should provide unprecedented breakthroughs in the study of individual variation in animal behaviors and the physiological costs associated with differing behavioral and life-history strategies.

In this theme issue, we examine the mechanistic underpinnings of individual variation in behavior and, specifically, how the physiological capacity for physical activity or “exercise” may directly enhance individual fitness. Aside from the direct biological implications of how activity is relevant in ecology, we also consider whether traditional methodological approaches and paradigms are appropriate for answering these questions. Though exercise is traditionally viewed as a strictly human endeavor (Van Dijk and Matson 2016), we argue that viewing various behaviors in non-human animals as analogous to exercise in humans will help us better understand the mechanisms underlying individual variation in traits as well as our methodological ability to measure performance accurately. Throughout this article, we consider movement and exercise broadly as any behavior that elevates the level of intensity of activity, in response to an ecological demand for increased performance. The papers in this issue span a range of animal taxa (including humans), types of activity, behavior or performance, ecological contexts, and include both laboratory- and field-based studies. Common themes which are addressed include: (1) individual variation in the level of behavior or performance, in response to challenging ecological scenarios; (2) physiological mechanisms underlying this individual variation; and (3) fitness consequences of this individual variation. The studies in this issue also broadly address the following four key questions relevant to the study of individual variation in activity in ecology.

## How hard do animals work in response to ecological (or evolutionary) demands?

**Fig. 1:**
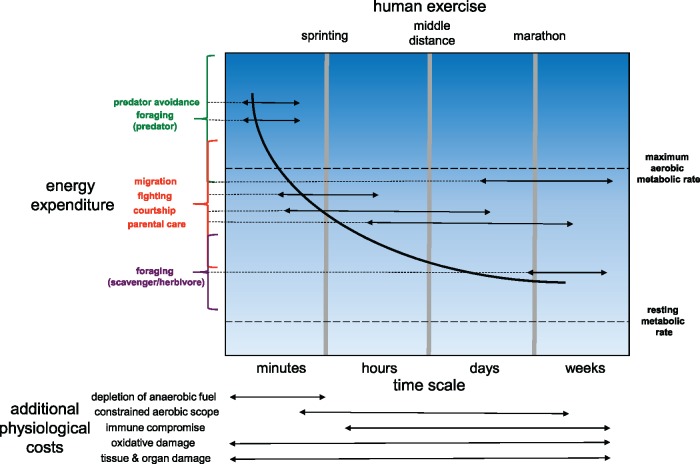
The potential costs of various behaviors associated with high levels of physical activity, inspired by Piersma (2011) and Peterson et al. (1990). The dark curve represents the sustainable level of energy throughput to support activity over a given time frame. At shorter temporal scales, increased energy can be spent on activity without incurring additional physiological costs. Activities that use amounts of energy above this line will potentially incur additional physiological costs (as indicated at the bottom of the figure) with potential implications for individual fitness. Individuals may minimise costs by either: (1) reducing the energetic costs of each behavior, by decreasing the frequency of each behavior or increase the efficiency with which it is performed; (2) adjusting physiological traits to attenuate the negative effects of operating above a sustainable level for various amounts of time (e.g., through training-induced plasticity). The width of the arrow associated with each type of behavior approximates the time scale over which each can occur; the elevation along the *y*-axis (in combination with the brackets along the *y*-axis) approximates the energy required for each behavior. Similarly, the width of the arrow associated with each physiological cost roughly indicates the temporal scale and activity types most likely to elicit each effect. At the top of the figure, types of human exercise (associated with running, specifically) are indicated that may be viewed as analogous to the non-human animal behaviors that elicit various intensities of activity at various temporal scales. Note that human exercise labels do not strictly align with the temporal labels along the *x*-axes.

Some activities of free-living animals are widely considered to be energetically-demanding “hard work,” such as long-distance migration (McWilliams et al. 2004; Farrell et al. 2008b). However, lower intensity activities associated with routine foraging, escaping predators (or potential mates), engaging in mating displays, and the provisioning of parental care also involve physical effort and can also be extremely costly (Fig. 1)(Bennett and Houck 1983; Nilsson 2002; Killen et al. 2007; Killen et al. 2016; Brownescomb et al. 2017; Garland et al. 2017). As a consequence, classical models in behavioral ecology—for example, those examining optimal foraging theory and risk-sensitive foraging—have included energetic trade-offs associated with the balance between foraging success (i.e., energy intake) and predator avoidance (Pyke 1984; Irschick and Garland 2001). The concepts underlying these models have proved versatile for examining the importance of individual variation in physiological “state” (e.g., variation in nutritional history or metabolic rate) for ecological phenomena (McLaughlin 1989; Killen et al. 2007; Farwell and McLaughlin 2009;). However, evolutionary fitness may be directly linked with the ability to engage in physical activity at sustained rather than maximum levels, with minimum physiological cost to tissues and organ systems (Piersma 2011), instead of the optimization of energy intake. Individual variation in the ability to withstand bouts of intense exercise and energy expenditure with direct and indirect fitness costs has been largely overlooked. It is also possible that physiological plasticity within the lifetime of individuals may modulate the costs of behaviors (see section discussing training, below).

As a result, we still have relatively little understanding of the “currency” by which ecological trade-offs are evaluated from an evolutionary perspective. Do animals actually prioritize the optimization of energy intake, or is this consideration modulated by other direct or indirect costs of physical activity? In addition to measuring the energy expenditure of individual animals while performing physical activity associated with parental care (Nilsson 2002; Cooke et al. 2006), foraging (Pontzer and Wrangham 2004; Killen et al. 2007; Williams et al. 2014), dominance contests (Killen et al. 2014; Seebacher et al. 2013), migration, and courtship (Ward et al. 2003; Woods et al. 2007), researchers now also frequently consider the costs of activities in terms of the maximal aerobic capacity (i.e., the factorial or aerobic scope) achievable by an animal and the proportion of this capacity that is occupied by an activity (Farrell et al. 2008a; Killen et al. 2016). Furthermore, more subtle differences in the costs of exercise experienced by individuals or species at the biochemical level may provide additional insight into the determinants of winners and losers in predator–prey scenarios (e.g., the proportions of carbohydrate versus lipid versus protein used as fuel types during physical activity; McClelland et al. 2017). A predator and its prey may be traveling at the same absolute speed during a pursuit, for example, but if one is operating closer to its capacity for aerobic metabolism then it may rely more heavily on carbohydrates for fuel during the chase, as opposed to more efficient lipid metabolism.

## Do lab-based studies of activity provide good models for understanding activity in free-living animals?

As discussed by Yap et al. (2017), a major obstacle in our efforts to understand the costs of physically demanding behaviors has been a reliance on laboratory measures of animal activity. Methods using treadmill running, wind-tunnel flying, and flume swimming are all largely divorced from ecological context, particularly because all of these scenarios simulate linear directional movement, while in reality, animals perform turns and bouts of acceleration that alter their costs of movement (Wilson et al. 2013). It is therefore reasonable to question the extent to which these methods for quantifying effort and the capacity for exercise are indeed appropriate for extrapolating conclusions to wild, free-ranging animals (Bidder et al. 2017). Even for humans, there is suggestion that established lab-based protocols may be insufficient for measuring maximum levels of oxygen uptake during exercise (Beltrami et al. 2012). Another serious deficiency associated with forced exercise protocols is whether animal motivation causes underestimates in the capacity for physical activity, if individuals behaviorally “choose” to cease exercising before they reach their physiological limit. Indeed, quantification of maximum oxygen uptake in humans during treadmill tests is believed to be strongly affected by psychological motivation to continue increasing activity to the peak levels that are physiologically possible (Noakes 2011, 2012; Beltrami et al. 2012). Variation in motivation to perform activity during a test could lead to spurious estimates of individual variability in traits related to maximum aerobic capacity or locomotor performance assumed to be attributable to physiological factors alone. Furthermore, stress experienced during laboratory estimates of energy expenditure may increase estimates of energy expenditure attributable to physical activity *per se* (Murray et al. 2017). There is also a question of whether benchmarks evaluated during lab tests such as maximum aerobic and anaerobic speeds and gait transition speeds are indeed ecologically relevant (Plaut 2001; Wolter and Arlinghaus 2003; Fisher et al. 2005).

The current wave of research quantifying the costs of ecologically relevant behaviors has been facilitated by technological advances that allow quantification of movement and energy expenditure at spatial and temporal scales that were previously impossible. In the lab, developments in respirometry equipment and in particular the widespread availability of optodes for measuring dissolved gases has revolutionized the measurement of metabolic rates and energy expenditure in aquatic animals (Svendsen et al. 2016). Methods for automated tracking of animals in behavioral arenas, including multi-agent tracking, have also allowed researchers to precisely measure activity levels of animals in laboratory experiments (Dell et al. 2014; Pérez-Escudero et al. 2014). There are also recent examples of researchers attempting to design methods for eliciting exercise in experimental settings, which are more ecologically relevant, such as requiring animals to exercise to obtain food items or access desired shelter or structure (Costantini et al. 2013). Tobalske et al. (2017) describe how the quantification of wing flapping during descent can be used as an ecologically relevant metric to help understand the ontogeny of flight muscle development in birds.

Perhaps the most important advances, however, have been in the realm of biotelemetry and remote sensing (e.g., geolocators, GPS, accelerometers) which are giving biologists an unprecedented ability to track movements and to understand inter-individual variability in the behaviors of free-living animals (Cooke et al. 2013a). One particularly exciting possibility here is to take animals into captivity, manipulate them (e.g., with specific diets), test them for physiological traits in the laboratory, and then release them with transmitters to experimentally study migration and other forms of activity (Baktoft et al. 2016). These technological developments are therefore allowing researchers to directly address questions of individual variation, mechanisms, and fitness consequences of variation in movement in novel ways (Sergio et al. 2014).

## Can animals work too hard during “routine” activities?

For any long-lived animal that is likely to experience more than one annual cycle, life-history theory predicts that individuals should rarely work so hard at any single activity (reproduction, migration) that they kill themselves, for example, individuals should invest more in self-maintenance, decreasing investment in current reproduction, if this allows them to maintain higher future fecundity and survival (Stearns 1992; Harshman and Zera 2007). Nevertheless, high intensity exercise such as migration, where animals operate at metabolic scopes of 8–15× their basal metabolic rate (BMR) over several days (Piersma 2011), can be associated with increased mortality (Newton 2006; Hinch et al. 2012; Klaassen et al. 2014) as well as reduced future fecundity (Fenkes et al. 2016; Smith and Moore 2003) in birds and fish. Furthermore, even sustained activity at lower intensity (2–4× BMR) is widely assumed to be sufficient to incur “costs” in some situations. For example, “cost of reproduction,” a key concept in life-history theory, describes the negative effect of current reproductive effort (e.g., foraging, chick provisioning during parental care) on future fecundity and survival (Daan et al. 1996; Williams 2012). In some species, animals direct so much time and energy to reproduction during the mating season that they die as a result (Bradley et al. 1980; Hinch et al. 2012), though it can be difficult to discern whether this mortality is due to changes in energy allocation, an increase in strenuous activity, a reduction in foraging to meet maintenance requirements, or a combination of these factors. Animals might also incur more subtle costs of activity, such as reduced foraging opportunities or decreased predator avoidance, if they have to recover from non-lethal costs of intense exercise. However, it remains largely unknown if free-living animals can really work “too hard” during a wide range of other routine activities such as patrolling territories, searching for mates, escaping predators, etc., and how important this might be for fitness.

The behavioral and physiological mechanisms underpinning costs of activity remain poorly understood (Harshman and Zera 2007; Williams 2012), especially for sustained, lower intensity activity. In humans, although increased physical activity can increase the endogenous production of reactive oxygen species and resultant oxidative damage of DNA and tissues, regular training appears to attenuate these negative effects, perhaps by increased oxidative defences or rate of tissue repair (Miyazaki et al. 2001; Powers et al. 2011). Whether non-human animals display similar responses to physical activity has not been thoroughly studied, but increased acute bouts of strenuous activity or prolonged exercise during migrations could increase oxidative damage for animals that are relatively inactive during other periods (Costantini et al. 2007; Monaghan et al. 2009). Given the debate about the universality of the energetic costs of behaviors associated with an increase in activity and the effects on life-histories (Shutler et al. 2006), identifying mechanisms allowing individuals to tolerate negative effects of bouts of intense activity (i.e., avoiding physiological costs beyond energy use) might be as important as identifying mechanisms that mediate costs leading to decreases in future fitness (Williams and Fowler 2015).

The obvious experimental approach to examine negative consequences of activity or workload is to make animals work harder and measure effects on fitness in terms of current reproduction, future fecundity, or survival. Laboratory studies of captive animals can achieve this using forced exercise paradigms, and these studies have reported negative physiological effects of intense exercise that would be consistent with “costs” (Yap et al. 2017). In the field, making animals work hard enough to show “costs” is much more problematic if individuals actually make “strategic” behavioral decisions: they might “choose” not to increase current workload in response to an experimenter-induced challenge, to preserve future fitness, or compensate by putting less effort into another type of behavior. A common technique is therefore to add weights to animals (Wright and Cuthill 1989), increasing body mass, or to reduce the size of locomotor structures, for example, wing clipping in birds (Rivers et al. 2017) and tail manipulation in fish (Basolo and Alcaraz 2003). An intriguing natural corollary of this experimental approach involves effects of large ectoparasites on swimming performance and fitness in fish (Binning et al. 2013; Binning et al. 2017).

Numerous studies using these direct, experimental manipulations of workload have looked for immediate, short-term effects (e.g., on the current breeding attempt) but few have comprehensively measured longer-term effects on future fecundity and survival. Furthermore, few experimental studies have insofar been coupled with detailed analysis of animal behavior using advanced bio-tracking technology which is now available. More detailed analysis of activity might therefore reveal complexities of behavior that help rationalize the often contradictory results of studies of “costs.”. As an example, many studies fail to find that wing-clipped birds reduce parental effort based on observed nest visit rates. However, measurement of overall activity (*sensu*Ward et al. 2014) using an automated telemetry array suggests that wing-clipping causes a significant decrease in the component of total “activity” directed toward self-maintenance (but not parental care measured as nest visit rate), and this was related to lower return rate (M. Serota and T. D. Williams, unpublished data). Thus, advances in bio-tracking technology will not only help us understand specific behavioral mechanisms related to costs of specific activities performed by individual animals, or avoidance of these costs, but will also (hopefully!) incorporate analysis of the role of biochemical, physiological, and morphological mechanisms of these costs associated with movement ecology.

## Can paradigms of “exercise” and “training” be applied to free-living animals?

Given the potential for routine locomotor costs and constraints on performance to affect individual fitness, it is worth considering how established frameworks for studying exercise physiology in humans may be useful for understanding ecophysiology in non-human animals (Halsey 2016). In this special issue, for example, Thompson et al. describe the physiological traits that limit performance in human athletes that engage in sprinting, middle-distance, and marathon running. These forms of athleticism have intriguing parallels with burst-type locomotion (as occurs during predator–prey interactions) and feats of endurance (e.g., migration) in ecology (Fig. 1), and may provide insight into how phenotypic variation in traits related to oxygen supply, muscular function, and neuroendocrine systems may directly determine performance and influence fitness in an evolutionary context. Interestingly, human performance during triathlons may provide a framework for understanding trade-offs experienced by animals adapted for locomotion in water and on land, or that are specialized for aquatic or terrestrial life at different times during their ontogeny (Calsbeek et al. 2017). The types of metabolic fuel (e.g., carbohydrates, lipids, and proteins) that are used during specific types of locomotor activity have also been extensively studied in humans (Talanian et al. 2007). As discussed by McClelland et al. (2017), variation in fuel use among species or individuals may also underlie variation in the costs of physical exertion of animals in different environments. The study of how psychological motivation constrains peak physical performance in humans (Noakes 2011, 2012)—as a buffer against complete physiological exhaustion—may provide insight into the degree to which motivation limits our ability to accurately measure maximum performance in non-human animals in laboratory tests (Thompson et al. 2017; Yap et al. 2017). Another major gap in our understanding of how exercise is relevant in animal ecology is the role of recovery after intense physical activity. Inter-individual variation in the ability to recover after strenuous exercise has been observed in humans, but we still know very little about individual variability in the capacity for recovery in non-human animals, the relationship between recovery ability and other physiological and behavioral traits, and the ecological relevance of recovery after exercise. Presumably, individuals that recover faster after agonistic interactions, predator–prey interactions, or migrations would have an advantage because they could resume regular activities sooner. In juvenile ambon damselfish, for example, individuals that are more aggressive and that have a higher aerobic scope return to normal levels of aerobic metabolism more quickly after fighting for territory with conspecifics (Killen et al. 2014).

Employing an exercise paradigm to ecological questions may also provide new perspectives on the constraints that animals face and the resources (i.e., time and energy) that they invest to overcome such limitations. For example, it is well known that humans must perform regular physical activity to maintain peak physical performance or capacity for endurance. Given that the consequences of under-performing are a matter of life and death for free-living animals—unlike human athletes at the Olympics, or couch potatoes—do other animals also need to engage in training to maintain their performance level or prepare themselves for demanding activities ((Halsey 2016); Bidder et al. 2017; Hawkes et al. 2017)? Routine levels of activity may be insufficient to prepare an individual for peak performance during more critical periods, such as during migrations or escaping a predator. Furthermore, if having the cellular machinery for increased peak performance is costly, then animals should be able to gain and lose that capacity for when it is and is not needed. In general, this research area is understudied, but it has been shown that repetition of behaviors in animals can lead to physiological changes that improve performance. These training effects relate directly to physiological plasticity and may directly affect the ability to withstand stressors to affect fitness. Bouts of intense activity over time may also have long term effects on behavior (i.e., personality; Sinclair et al. 2014). This type of plasticity may be very important in an evolutionary context as well, not only generating phenotypic variation among individuals, but also allowing animals to reduce the energy costs of activity by increasing the efficiency of locomotion or heightening the ceiling limiting peak levels of performance (Killen et al. 2016). Training-induced plasticity is also observed in human athletes, which show increased economy of movement when compared to people that are untrained (Jones and Carter 2000; Joyner and Coyle 2008). Amazingly, however, there is evidence that migratory birds can undertake incredible feats of endurance with no apparent change in behavior or training in the lead up to migration (Portugal et al. 2011; Hawkes et al. 2017). Increased physical activity may also alter the effectiveness of the immune system in wild animals. In humans, exercise has been shown to have complex effects on immune system indices that appear related to the intensity and duration of the activity performed (i.e., acute exercise versus prolonged training), though the exact mechanisms underlying changes in immune function brought on by exercise are not well-understood, even for humans (Pedersen et al. 1998; Pedersen and Hoffman-Goetz 2000; Nieman 2003; Van Dijk and Matson 2016)

## Conclusions

We suggest that the study of behavioral ecology and ecophysiology will be enhanced by embracing the concepts of “exercise” and “training” as frameworks for understanding the locomotor constraints faced by animals in their natural environment. This approach will encourage the direct quantification of the energetic costs of behaviors related to fitness and allow us to appreciate how limitations beyond the optimization of energy input may influence individual variation in behaviors and the resulting evolutionary trajectories. Furthermore, viewing the behaviors of non-human animals as exercise will allow ecologists to take advantage of established knowledge and approaches (Nieman 2003; Joyner and Coyle 2008; Noakes 2011), already in use by human exercise physiologists, for identifying key traits that define performance and accurately measuring their influence. By highlighting mechanisms of behavior and performance throughout this issue, we hope to foster collaborations whereby physiologists and endocrinologists can work with ecologists, to fully exploit the potential of emerging cross-disciplinary perspectives and technologies for tracking the movements and physical activity of individual animals in the laboratory and in natural or semi-natural environments.
